# Data of co-extraction of inulin and phenolic compounds from globe artichoke discards, using different conditioning conditions of the samples and extraction by maceration

**DOI:** 10.1016/j.dib.2020.105986

**Published:** 2020-07-05

**Authors:** Carmen Soto-Maldonado, María Elvira Zúñiga-Hansen, Araceli Olivares

**Affiliations:** 1 Regional Center for the Study of Healthy Foods. Av. Universidad 330, Valparaíso, Chile. GORE-CONIYCT R17A10001; 2 School of Biochemical Engineering, Pontifical Catholic University of Valparaíso, Av. Brasil 2085, Valparaiso, Chile

**Keywords:** Inulin, artichoke discards, *Cynara scolymus* L., bioactive compounds extraction, vegetable sample conditioning

## Abstract

The current data presented correspond to the determination of inulin recovery from globe artichoke canned industry wastes. The discard was composed mainly by bracts with a small percentage of stems and receptacles. Artichoke discards (AD) were dehydrated by lyophilization or convective drying at different temperatures (40°C to 100°C). Inulin amount in extracts obtained using hydroalcoholic solvents (ethanol:water 75:25), which are applied for polyphenols recovery, was determined. After that, the sequential extraction of inulin with water and then with hydroalcoholic solvent was done. Finally, inulin content in lyophilized samples using different ethanol:water mixtures was determined. Inulin was determined by vanillin method and total phenolic compounds (TPC) by Folin-Ciocalteu method. From the lyophilized sample it is possible to obtain 3938.7 ± 169.1 mg _inulin_ / 100 g _AD_ dry basis (d.b.) and 2086.3 ± 120.7 mg _TPC_ / 100 g _AD_ d.b. While, from conventionally dried samples, the recovery of inulin can reach 4391.1 ± 208.2 mg _inulin_ / 100 g _AD_ d.b for samples dried at 60°C, but only 337.2 ± 25.9 mg _TPC_ / 100 g _AD_ d.b. was recovered at the same condition. Sequential extraction of lyophilized samples with water (95°C, 30 minutes) and ethanol:water 75:25 (40°C, 60 minutes) recovers in total 10907.3 mg _inulin_ / 100 g _AD_ and 2687.7 mg _TPC_ / 100 g _AD_ d.b. If the ethanol concentration decreases at 50% and the extraction is done only with the hydroalcoholic solvent, the inulin increases up to 5251.2 ± 257.4 mg _inulin_ / 100 g _AD_ d.b.

This Data in Brief corresponds to an accompanying work to the article titled “Valorization of Globe Artichoke (Cynara scolymus) Agro-Industrial Discards, Obtaining an Extract with a Selective Effect on Viability of Cancer Cell Lines” published at Processes journal [1].

## Specifications Table

**Table:** 

**Subject**	Process Chemistry and Technology
**Specific subject area**	Recovery of bioactive compounds, Valorization of agro-industrial waste
**Type of data**	Figures
**How data were acquired**	The presence of inulin and phenolic compounds were determined using spectrophotometric methods (see experimental design, materials and methods section). UV-visible spectrophotometer (Jasco, model v630) and a microplate reader (Thermoscientific, model Multiskan go) were used.
**Data format**	Raw, analyzed and quantified by calculation
**Parameters for data collection**	The factors taken into account to generate the data presented were: a) the conditioning of the artichoke discard raw material (lyophilization and conventional drying at different temperatures), b) concentration of ethanol in the extraction solvents using the lyophilized samples. The presence of inulin in extracts obtained by processes done with a focus on phenolic antioxidant recovery is determined.
**Description of data collection**	Data of inulin and phenolic compounds (TPC) were determined in extracts obtained from artichoke discards (mainly bracts) dehydrated by lyophilization or conventional drying at 40, 60, 80 and 100°C. Also, a sequential extraction was performed, first with water and then with the hydroalcoholic solvent on the residual solids, obtaining data of inulin and TPC. Finally, inulin data was obtained, changing the proportion of ethanol in the solvent using lyophilized samples. In all cases, the extraction temperature was kept constant, and the supernatant obtained by vacuum filtration.
**Data source location**	The artichoke discards were obtained in a canned industry in the town of San Felipe, Valparaíso Region (32°44′59.1′' S 70°43′33′' W), Chile. The extraction and analyzes were carried out in the city of Valparaíso, Valparaíso region (33°1’47.35” S 71°37’40.91”W) Chile in the institution: Centro Regional de Estudios en Alimentos Saludables (Regional Center for the Study of Healthy Foods)
**Data accessibility**	With the article. Raw data are presented in supplementary document (each sheet in the excel file, as they are named, is associated with a Figure presented in the Data description section)
**Related research article**	Noriega-Rodríguez, D.; Soto-Maldonado, C.; Torres-Alarcón, C.; Pastrana-Castro, L.; Weinstein-Oppenheimer, C.; Zúñiga-Hansen, M.E. Valorization of Globe Artichoke (Cynara scolymus) Agro-Industrial Discards, Obtaining an Extract with a Selective Effect on Viability of Cancer Cell Lines. Processes 2020, 8, 715. https://doi.org/10.3390/pr8060715

.

## Value of the Data

• The data provide information about the co-extraction of inulin and phenolic compounds present in globe artichoke (*Cynara scolymus* L.) discards, using hydroalcoholic solvents.
• Knowing the presence of such compounds allows generating alternatives for the valorization of these discards that leads to economic and environmental benefits due to 1) avoiding the disposal of agro-industrial discards; 2) introducing two valuable bio-products with potential application in different industrial sectors (food and nutraceutical industries).
• Also, the data is the starting point to establish different biological activities of the extract, to evaluate potential synergistic effects (or not) of both types of compounds present in it, and of fractionation processes that improve the quality of the final product.
• The data shows that the process conditions affect the extract composition, about the content of inulin and phenolic compounds with antioxidant activity [1], [2], [3], [4], promoting the development of a new product with potential health benefits. Furthermore, as they originate from agro-industrial discards, the environmental impact of this business sector is reduced.

## 1 Data Description

The presented data include the quantification of inulin and total phenolic compounds in extracts obtained from globe artichoke (*Cynara scolymus* L.) discards from a canned industry. The extraction was using hydroalcoholic solvents. Raw data are in the supplementary material.

**Fig. 1:**
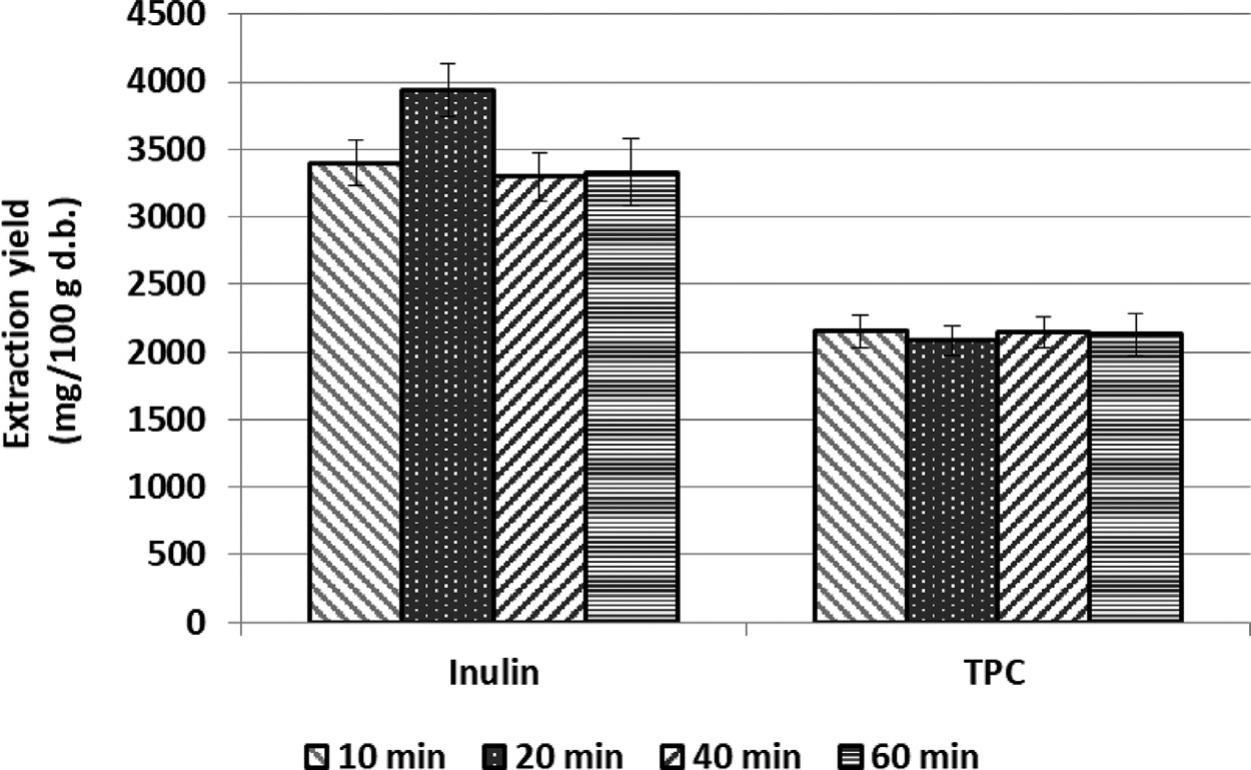
Recovery of inulin and total phenolic compounds (TPC) from lyophilized samples of artichoke discard (mainly bracts). Extraction conditions: 40°C, ethanol:water 75:25, solid/solvent ratio 1/20. Data expressed as mg of compound per 100 g of dehydrated artichoke discard. Data represented the mean value ± Standard deviation (SD) (*n* = 3).

Fig. 1 shows the recovery of both inulin and phenolic compounds from the artichoke discards when they have been lyophilized. The lyophilization process takes place under mild temperature conditions (-50 to -20°C), so it is used for raw materials that contain compounds with biological activity.

**Fig. 2:**
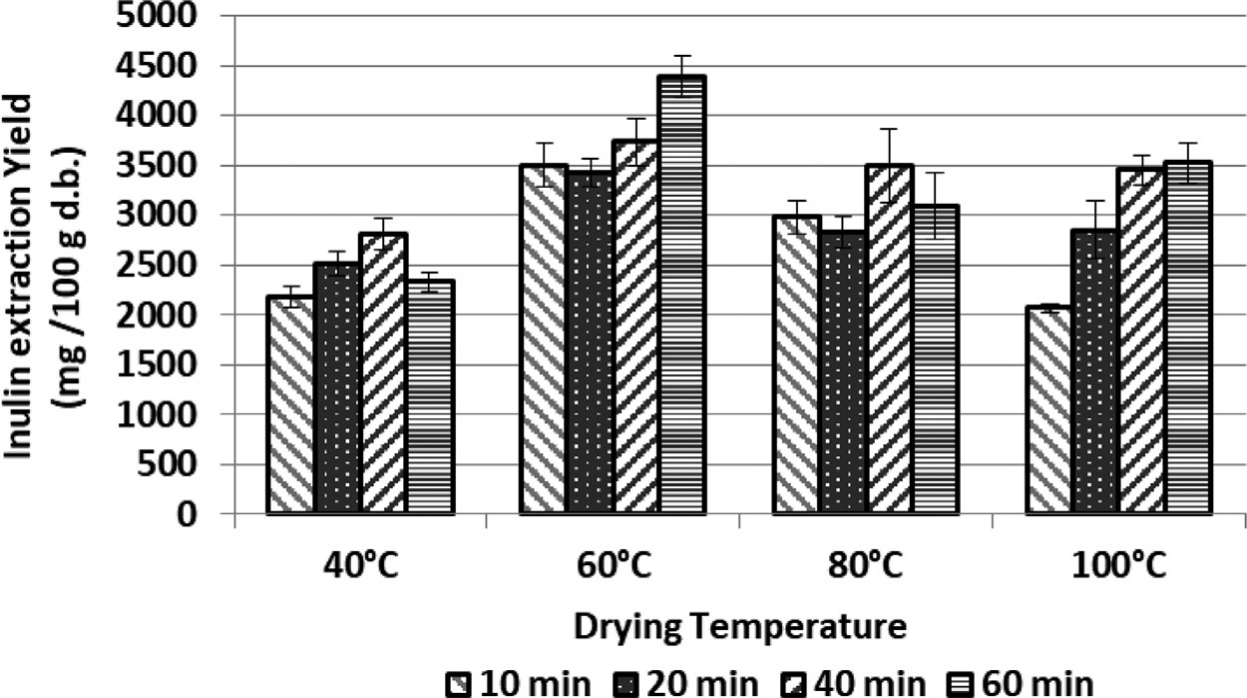
Recovery of inulin from samples of artichoke discard (mainly bracts) dried in a conventional oven at different temperatures. Extraction conditions: 40°C, ethanol:water 75:25, solid/solvent ratio 1/20. Data expressed as mg of compound per 100 g of dehydrated artichoke discard. Data represented the mean value ± SD (*n* = 3).

**Fig. 3:**
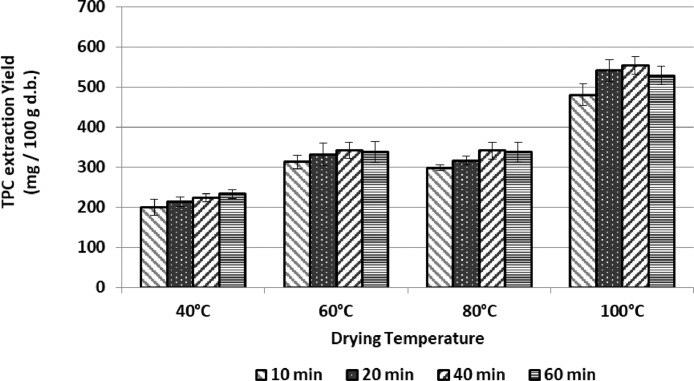
Recovery of total phenolic compounds (TPC) from samples of artichoke discard (mainly bracts) dried in a conventional oven at different temperatures. Extraction conditions: 40°C, ethanol:water 75:25, solid/solvent ratio 1/20. Data expressed as mg of compound per 100 g of dehydrated artichoke discard. Data represented the mean value ± SD (*n* = 3).

Figs. 2 and 3 shows how the recovery of inulin and total phenolic compounds varies from artichoke discards dried in a convective stove at temperatures from 40 to 100°C.

**Fig. 4:**
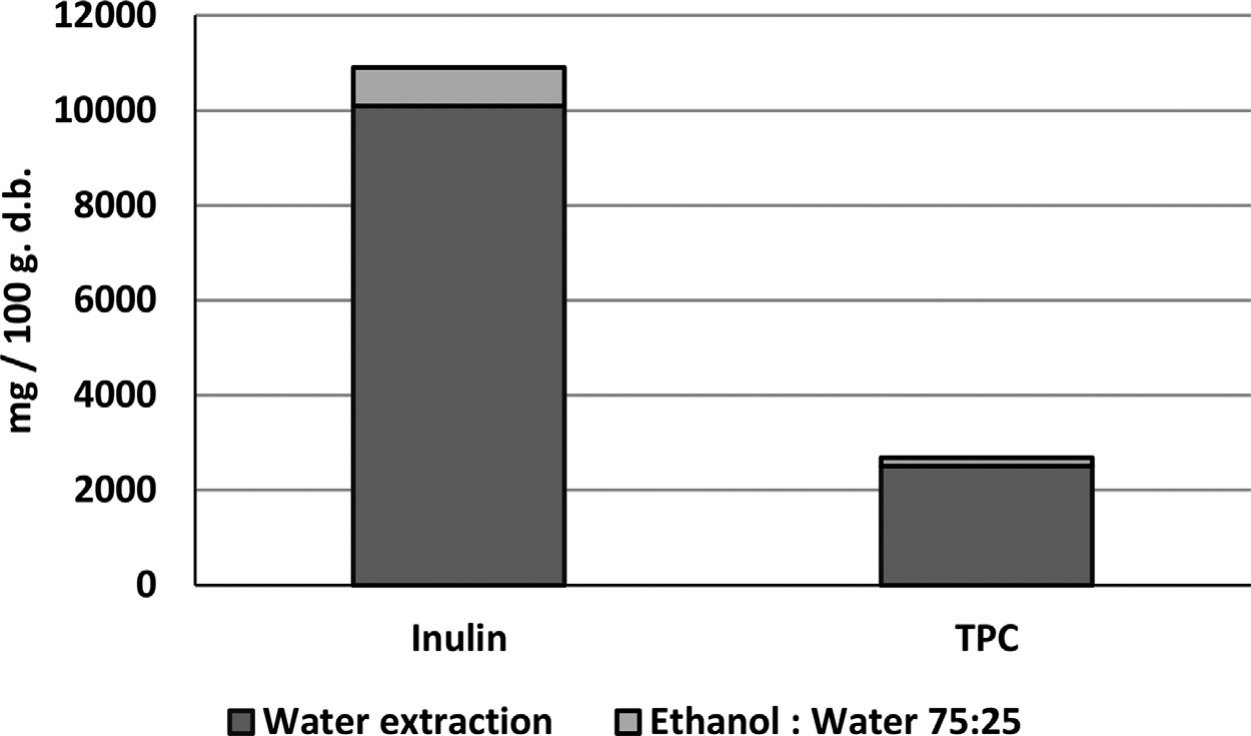
Recovery of inulin and total phenolic compounds (TPC) from lyophilized samples of artichoke discard (mainly bracts). Extraction conditions: sequential extraction using water (95°C, 30 minutes); and ethanol:water 75:25 (40°C, solvent/solid ratio 1/20, 60 minutes). Data expressed as mg of compound per 100 g of dehydrated artichoke discard. Data represented the mean value ± SD (*n* = 3).

Lyophilized samples of artichoke discards extracted with water, and the residual solids extracted with a hydroalcoholic solvent, produce extracts with inulin and phenolic compounds in both fractions (Fig. 4).

**Fig. 5:**
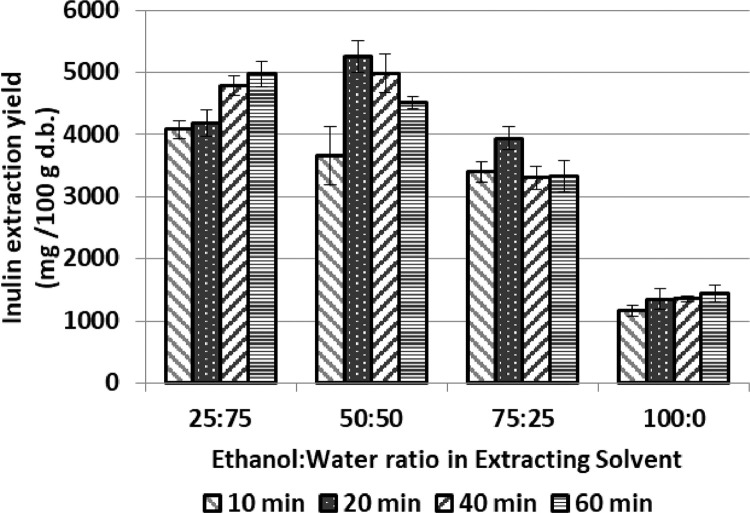
Recovery of Inulin (TPC) from samples of lyophilized artichoke discard (mainly bracts) using several proportions of ethanol in the extracting solvent. Extraction conditions: 40°C, solid/solvent ratio 1/20. Data expressed as mg of compound per 100 g of dehydrated artichoke discard. Data represented the mean value ± SD (*n* = 3).

Fig. 5 shows the recovery of inulin from lyophilized artichoke waste samples, using different ethanol-water mixtures. The extraction obtained with ethanol:water 50:50 was higher than only with ethanol.

## 2 Experimental Design, Materials and Methods

Artichoke discard was obtained from a canned industry in the city of San Felipe (Valparaíso Region, Chile).

The discard is composed mainly by bracts (95%) and a small number of stems and receptacles. The raw material was dehydrated using lyophilization or convectional convective drying. In the first case, the process was carried out using an Operon FDT 8632 Freeze Dryer, while the convective drying was done in a Memmert UFE 550 stove, using 40°C, 60°C, 80°C and 100°C. The samples are dehydrated until reaching a humidity equal to or less than 5%. Then, the samples were milled using an IKA A11 basic mill, to obtain a particle size less than 0.5 mm.

First, the extractions were assayed with 1 g of the sample and 20 mL of the extracting solvent. The temperature was 40°C in an orbital incubator. The extraction solvent was a hydroalcoholic mixture using ethanol (ethanol: water 75:25). This mixture is used for obtaining phenolic compounds.

After the extraction time, the supernatant was recovered by vacuum filtration using Whatman n°1 filter paper. The extracts were freeze stored until its analysis.

Sequential extraction of the solids was evaluated. Initially, lyophilized artichoke discard samples were extracted with water at 95°C, 1/50 solid/liquid ratio, 30 minutes; the supernatant recovered, and the solids re-extracted with the hydroalcoholic solvent under the previously mentioned conditions for 60 minutes. The presence of inulin and total phenolic compounds were determined in both supernatants.

The recovery of inulin and phenolic compounds from lyophilized samples of artichoke discard was also determined, using different hydroalcoholic mixtures (ethanol: water 25:75, 50:50, 75:25) and only ethanol for extraction.

Inulin content of the extracts was determined with the vanillin method proposed by Levine and Becker (1959) [5]. Briefly, the extract was deproteinized as follow: 1 mL of sample, 5 mL of Milli-Q water, 2 mL of ZnSO_4_⋅10 H_2_O 10%, and 2 mL of NaOH 0.5N were mixed and filtrated. Then, a mixture of 1: 2.5: 2.75 of vanillin 1%: filtrate: sulfuric acid was done. The mixture was shaken, boiled for 2 minutes, cooled, and left to stand for 15 minutes at room temperature. The absorbance at 520 nm was recorded using a Jasco (model v630) UV-Vis spectrophotometer. Commercial inulin was used as standard. The data expressed as mg of inulin per 100 grams of dehydrated artichoke discard. Total Phenolic Compounds (TPC) of the extract was determined, according to Soto *et al*. (2014)[6]. Briefly; 24 μL of the sample, 180 μL of distilled water, 12 μL of Folin-Ciocalteu reagent 50% and 24 μL of sodium carbonate 10% were in a 96 deep well plate. The mix was left to stand at room temperature of 1 hour. Then, the absorbance at 765 nm was recorded using a Thermoscientific (model Multiscan go) microplate reader. Gallic acid was used as standard. The data presented as mg of gallic acid equivalent per 100 grams of dehydrated artichoke discard.

## Ethics Statement

In this research, no work with animals or humans

## Author's Contribution

**Carmen Soto-Maldonado:** Conceptualization; Funding acquisition; Investigation; methodology; resources writing-original draft and review & editing

**María Elvira Zúñiga-Hansen:** Conceptualization; Funding acquisition; resources

**Araceli Olivares – Miralles:** Investigation, validation, resources, writing-original draft and review & editing.

## Declaration of Competing Interest

The authors declare that they have no known competing financial interests or personal relationships which have, or could be perceived to have, influenced the work reported in this article.

## Appendix Supplementary materials

Image, application 1

Image, application 1
